# Fully Virtual, Focused Applied Behavior Analysis Services: Acceptability and Feasibility Study

**DOI:** 10.2196/90243

**Published:** 2026-06-10

**Authors:** William H Edwards, Brittany C Wierzba

**Affiliations:** 1 Center for Behavior Analysis, Clemson University, B-111 Barre Hall, Clemson, SC, 29634, United States, 1 864-656-0953; 2 AnswersNow, Inc, Richmond, VA, United States

**Keywords:** applied behavior analysis, comprehensive treatment, focused treatment, autism spectrum disorder, Board Certified Behavior Analyst

## Abstract

**Background:**

Traditional models of applied behavior analysis (ABA) services for those diagnosed with autism spectrum disorder (ASD) involve time-intensive, labor-intensive, comprehensive, in-person services. The increasing prevalence of ASD in the United States has precipitated a need to identify effective, accessible, and innovative methods to address the needs of those with ASD.

**Objective:**

The present descriptive study examines the feasibility and acceptability of a fully virtual, targeted model of ABA service delivery.

**Methods:**

This retrospective analysis evaluated changes over time in adaptive behavior, maladaptive behavior, and caregiver satisfaction during focused, virtual ABA therapy delivered by a Board Certified Behavior Analyst.

**Results:**

Improvements in standard scores across Vineland-3 domains and improvements in quality of life (Child and Family Quality of Life, Second Edition), in addition to high levels of caregiver satisfaction, were observed over time.

**Conclusions:**

While causality cannot be determined for these improvements, results support the feasibility and acceptability of this novel ABA service delivery model.

## Introduction

Behavior analytic services can have profound positive impacts on individuals and their families. Behavior analysts identify behaviors to teach or reduce by working closely with stakeholders and use this information to select approaches for analyzing and changing behavior, and ultimately, to create socially significant and meaningful change for individuals [1]. As social significance varies from person to person, environment to environment, and culture to culture, the specific behaviors targeted by behavior analysts can be as varied as vocational skills and dental care, shoe tying and aggressive behavior, or law enforcement personnel behaviors and kindergarten readiness skills [2].

Due to efforts driven by families, grassroots organizations, service providers, and researchers across several decades, behavior analytic services are widely implemented to support autistic individuals and their families [3] (In an effort to respect the diverse perspectives of those on the autism spectrum, we use both identity-first language [eg, “autistic child”], person-first language [eg, “child with autism”], and neutral language [eg, “person on the autism spectrum”] to describe our participants and the recipients of applied behavior analysis [ABA] therapy [4]). According to a review conducted by Gitimoghaddam et al [5], ABA has been recognized as one of the most empirically validated forms of support for persons diagnosed with autism spectrum disorder (ASD). In recent years, the prevalence of ASD has become a topic of national interest within the United States, particularly following the release in April 2025 of updated prevalence data from 2022. This report indicates an increase in prevalence to 1 in 31 children from 1 in 36 children in 2020 [6].

As of 2019, all 50 states in the United States require “meaningful coverage” for autism treatment, including ABA therapy services [7]. When services are covered by medical insurance, providers must demonstrate medical necessity and clinical progress [8], both of which require the assessment of current functioning over time. Despite the widespread nature of insurance mandates and the common need to evaluate current functioning, there is limited consensus about the best measures to evaluate clinical outcomes or response to treatment [9,10]. Two measures identified to include in the ICHOM’s (International Consortium for Health Outcomes Measurement) Standard Set of outcomes for ASD are the Vineland Adaptive Behavior Scales-Third Edition (Vineland-3) and the Child and Family Quality of Life, Second Edition (CFQL-2) [9].

The Vineland-3 assessment measures caregiver-reported adaptive and maladaptive behavior [11] and has been used as an outcome measure in a variety of studies evaluating services for autistic individuals [12-15]. One such study evaluated changes in both the adaptive and maladaptive domains from the Vineland-3 as a function of the client’s ASD severity level and usage of prescribed therapy hours [14]. The authors stratified participants by age at the first Vineland-3 administration as well as gender and analyzed changes in the adaptive domains and the extent to which participants’ maladaptive subdomain scores improved from clinically significant to nonclinically significant following treatment. This approach of including Vineland-3 maladaptive subdomain score changes is not widespread in the ABA therapy outcomes literature, highlighting an important gap in the literature.

Although specific thresholds for minimal clinically important differences (MCID) have not yet been established for the Vineland-3, previous studies, such as Ostrovsky et al [12], have compared their participants’ standard score changes over time, with MCID thresholds derived from MCIDs established for the Vineland-II assessment [16]. The MCID thresholds used by Ostrovsky et al [12] were +2.0 for the ABC (Adaptive Behavior Composite) standard score,+2.0 for the communication standard score,+2.6 for the socialization standard score, and +2.6 for the daily living standard score. While these score changes reflect an anchor-based approach to establishing clinical significance, they do not reflect industry data from actual clinical practice. Despite the lack of agreement within the industry about which tools are most appropriate and how to evaluate meaningful change, there is consensus that teaching adaptive behaviors is a primary goal of ABA services [17].

Some research shows a relation between adaptive skills and quality of life (QoL) for individuals with intellectual disability, with stronger adaptive skills being correlated with improved QoL [18]. Among autistic adults, factors associated with QoL may be even more complex than within other adult populations, making the inclusion of this measure critical to understanding how interventions can support improved QoL [19]. Given the interconnected nature of different QoL areas within a family system, one way of evaluating QoL is by assessing the psychosocial aspects at the individual and family level [20]. The CFQL-2 has been established as a reliable measure of psychosocial aspects of QoL, including child, family, caregiver, the financial, external support, partner relationship, coping, and QoL change [21]. Ciobanu et al [22] observed improvement in total QoL and across several subscales for a series of three patients receiving ABA therapy delivered by a parent behavior technician. These results suggest that, for the patients in their study, equipping parents with the knowledge and skills to support their own child’s behavior change may have a positive impact on QoL for the whole family system. The extant literature using the CFQL-2 to evaluate QoL among individuals on the spectrum is limited [22,23], but, given the inclusion in the ICHOM ASD standard set [9], this tool may gain traction among ABA service providers interested in evaluating client progress from a variety of angles. A threshold for clinically significant change has not yet been established.

Today, the predominant form of ABA therapy in the United States is center- or home-based services delivered in a 2- or 3-tier model, whereby a Registered Behavior Technician (RBT) is the primary deliverer of care and is directly supervised by a Master’s- or Bachelor’s-level behavior analyst (often a Board Certified Behavior Analyst [BCBA]). This model is typically considered “comprehensive,” which is defined as a scope of treatment that “typically encompasses multiple simultaneous goals within and across multiple domains, such as language, behavior, activities of daily living, social skills, and cognition” in their practice guidelines by the Council for Autism Service Providers [24]. According to these guidelines, comprehensive ABA treatment generally requires 30‐40 hours of therapy per week to ensure adequate time for teaching and practice opportunities.

Although there is evidence of the efficacy of in-person, comprehensive ABA therapy delivered in a 2- or 3-tier model [25], there are also many challenges related to this service delivery model. The first and most common challenge related to the traditional service delivery model is a lack of access due to geography or a lack of providers within proximity. According to the US Census Bureau census conducted in 2020, a total of 80% of the population of the United States live in urban areas (defined as greater than 2500 people), and 20% live in rural areas (defined as 2500 people or less). Additionally, the availability of services in general tends to be concentrated in larger metropolitan areas [26].

Families who do have access to ABA services within reasonable proximity of their homes may experience other challenges. First, the available services do not always meet the needs of specific autistic individuals. In particular, school-age children with less global functional impairment may not be well-served by a comprehensive treatment program whose time commitment impacts school attendance or participation in enriching extracurricular activities. Recent studies examining the efficacy of lower intensity ABA services have demonstrated support for less time-intensive interventions [13,27].

Next, the comprehensive model relies on lesser-qualified service providers (eg, a provider with a high school diploma and 40 hours of training, supervised by a Master’s- or PhD-level provider roughly 5% of the time) to deliver the bulk of client care. Turnover among these providers is high, with the Behavioral Health Center of Excellence reporting annual turnover of 65% in its 2022 ABA Compensation and Turnover Benchmark Report [28]. These high levels of turnover impact caregiver satisfaction [29] and may result in gaps in care or in lower-quality services being provided. According to current literature exploring parent training and caregiver-mediated interventions, increased caregiver participation in intervention can reduce parental stress levels, increase parental self-efficacy, and support positive child outcomes [30]; however, high levels of turnover and other barriers may prevent families from being meaningfully involved in care.

Finally, the costs associated with health care for a child with autism, including traditional ABA services, are significant. A recent report from the Agency for Healthcare Research and Quality indicates that the average total health cost for a child being treated for autism was US $20,122 compared with the average total health cost of US $2201 for a child not being treated for autism [31].

Given the challenges associated with the comprehensive ABA service delivery model, some providers are seeking alternative models that address the concerns detailed above while still producing meaningful change on behalf of the families they serve. One relatively new option is a BCBA-direct, fully virtual, focused ABA therapy model. Services delivered via telehealth are less constrained by the geographic locations of the client or clinician, making it possible for clinicians to reach clients in more rural areas, as well as those residing in areas with a lower ratio of providers to clients. A growing body of literature supports the use of telehealth as an ABA service delivery modality [32-34]. Another benefit of this approach is that individuals who have more targeted clinical needs can receive more tailored interventions, focusing on the area of functional impairment that is most impactful at the time of treatment. These services can be delivered at a lower “dosage” than the recommended 30‐40 hours per week for comprehensive services, potentially making them more accessible to busy families and more cost-effective for families and their funding sources. This approach is further bolstered by more frequent and consistent contact with a BCBA, who can make treatment decisions throughout each therapy session and evaluate procedures on an ongoing basis, unlike therapy models that rely on RBTs rendering the bulk of service hours. Finally, the telehealth treatment modality can allow for increased caregiver involvement and, therefore, a potential increase in naturalistic learning opportunities by removing many of the barriers families receiving center-based services encounter, such as logistical difficulty with going to the center for caregiver training sessions and a lack of generalization of skills being targeted in an environment very different from their home.

The purpose of this study was threefold: (1) to describe a novel ABA service delivery model that could serve as an alternative for some families; (2) to examine changes in adaptive skills, maladaptive behaviors, and QoL over time for participants who received this novel form of ABA therapy; and (3) to assess the social validity of this novel treatment model through a caregiver satisfaction survey. Evaluation of the fully virtual, BCBA-only, focused treatment model is important because this model represents a structurally different approach to delivering care compared to in-person, RBT-implemented, comprehensive therapy models and has the potential to mitigate many of the challenges associated with prevailing models.

## Methods

### Service Model

Behavior analytic services were delivered through a fully virtual, BCBA-only, focused treatment model provided by AnswersNow Inc, an ABA service provider. Clinicians delivered services through proprietary, HIPAA (Health Insurance Portability and Accountability Act)-compliant, synchronous audio-video software. All services were rendered by a BCBA, including assessment, direct therapy, therapy protocol modification, and caregiver training. At the outset of care, the assigned BCBA conducted a comprehensive assessment with each participant that included the Vineland-3, the CFQL-2, indirect behavior assessments (eg, FAST [functional assessment screening tool] and QABF [questions about behavior function]), and direct skill assessments based on the participant’s clinical presentation (eg, VB-MAPP [Verbal Behavior Milestones Assessment and Placement Program] and AFLS [Assessment of Functional Living Skills]). The BCBA synthesized assessment results to determine the participant’s strengths and areas of need, as well as to identify any behaviors that interfered with the participant being able to learn new skills or successfully contact less restrictive environments. The BCBA then developed goals to address the identified behaviors and selected behavior analytic intervention procedures to implement.

Following the initial assessment and throughout the course of treatment, the BCBA updated goals and adjusted therapy procedures in response to the participant’s progress. While the specific goals targeted and teaching procedures used varied and were individualized to each participant, all BCBAs prioritized behaviors that were meaningful for families and/or participants and implemented procedures consistent with the principles of behavior analysis. At 6-month intervals, the BCBA conducted a reassessment to evaluate clinical progress and adjust goals accordingly. This reassessment included readministration of the Vineland-3, the CFQL-2, indirect behavior assessments, and direct assessments based on the participant’s clinical presentation.

During ongoing treatment, participants received a combination of direct therapy, caregiver-mediated therapy, and caregiver training without the child present. The ratio of these different service types fluctuated throughout the course of treatment based on the participants’ and families’ needs; all families participated in caregiver training during the course of treatment, whether through caregiver-mediated services or caregiver training without the participant present, with a mean of 2.6 (SD 1.05) hours per week (range 0.25 total h to 6.37 h/wk). Direct therapy sessions consisted of the child interacting directly with the BCBA via synchronous audio-video, with screen-sharing capabilities. Caregiver-mediated sessions consisted of the child, caregivers, and BCBA being present via synchronous audio-video with the BCBA providing coaching and guidance to the caregiver, focusing on strategies for teaching new skills or reducing challenging behaviors, with the caregivers interacting directly with the child. Finally, caregiver training consisted of the BCBA and caregivers meeting via synchronous audio-video. During these sessions, the BCBA provided training to the child’s caregivers on specific interventions for teaching their child new skills or reducing challenging behaviors.

### Participants

Following IRB review and obtaining an exempt status determination, a sample of deidentified records from individuals who received ABA services through AnswersNow, Inc, between October 2021 and October 2025 was obtained. Participants were included if they had research consent on file, ASD listed as their primary diagnosis, at least two valid administrations of the Vineland-3 Comprehensive Parent/Caregiver Form (defined as the assessment being completed by the participant’s parent or caregiver, not the clinician), at least four months between the first and most recent administration, and at least 15 minutes of ABA therapy (irrespective of service type) per week between the first and most recent administrations. When possible, the same caregiver completed the Vineland-3 at each administration; however, there were instances within the dataset in which 2 different caregivers completed the first and most recent assessments.

### Ethical Considerations

AnswersNow, Inc, obtained consent to use deidentified clinical data for research purposes from all participants through a general research consent form signed by families either at the outset of treatment or during services. This research-specific consent was voluntary and revocable at any time, with no relationship to services rendered. Participant data were deidentified before analysis, and no personally identifiable information was shared with the researchers or used in this study. The Clemson University Office of Research Compliance determined the proposed activities involving human participants met the criteria for exempt level review under 45 CFR 46.104(d), and exempt level status was rendered on April 2, 2025 (IRB2025-0021). The data were encrypted and stored in accordance with the Clemson University Minimum Security Standards and analyzed using SPSS (version 31; IBM Corp).

### Procedures

The analysis involved evaluating change over time in the results obtained from the comprehensive battery of assessments administered every 6 months, beginning with the first administration until the last administration, regardless of the number of administrations. Any participant with only one administration was not included in the sample. The comprehensive assessment included the administration of the Vineland-3, including all adaptive domain scales (communication, socialization, daily living skills, and ABC), Vineland-3 maladaptive domain scales (ie, internalizing and externalizing) for a subset of participants, the CFQL-2 scales (child, family, caregiver, financial, social network, partner relationship, coping, and overall), indirect behavior assessments (eg, FAST and QABF), and direct assessments based on the participant’s clinical needs (eg, VB-MAPP and AFLS).

For the purposes of this study, the domain scale scores from the domains in the Vineland-3 assessment tool and the CFQL-2 assessment tool were used to assess participant progress and outcomes while receiving virtual, focused, BCBA-direct ABA therapy. Scale scores from each domain in the Vineland-3 and the CFQL-2 were captured from each administration. MCIDs were calculated for Vineland-3 score changes based on the thresholds used by Ostrovsky et al [12] (ie, +2.0 for ABC standard score, +2.0 for communication standard score, +2.6 for socialization standard score, and +2.6 for daily living standard score). Additionally, comparison analyses similar to those included by Adelson et al [14] were conducted to evaluate maladaptive behavior changes and to determine the potential influence on score change from several variables, including gender, insurer, hours of services, and ASD severity level.

## Results

### Sample Characteristics

A total of 504 participants from the original dataset of 1441 met the criteria to be included in this study. The total sample of 504 participants included 77% (388/504) males and 23% (116/504) females, and the average age at first Vineland-3 administration was 8.4 (SD 4.3; range 1‐22) years. Participants were located across five different states: California, Georgia, New York, Texas, and Virginia. Among the 3.8% (19/504) of participants located in California, 18 were Medicaid recipients and 1 participant was not. Participants located in Georgia represented 40.7% (205/504) of the total sample, 153 of whom were Medicaid recipients and 52 of whom were not. The 0.2% (1/504) of participants located in New York consisted of 1 Medicaid recipient. Among the 7.5% (38/504) of participants located in Texas, 25 were Medicaid recipients and 13 were not. Finally, most participants, 47.8% (241/504), were located in Virginia; among these participants, 155 were Medicaid recipients and 86 were not.

ASD severity levels were available for 29.6% (149/504) of participants. This information was not available for all participants because it was not provided by the diagnosing professional. Within the social communication diagnostic domain, 39.6% (59/149) of participants’ diagnostic reports indicated diagnostic severity level 1, 34.9% (52/149) indicated level 2, and 25.5% (38/149) indicated level 3. Within the restrictive and repetitive behaviors diagnostic domain, 40.9% (61/149) of participants’ diagnostic reports indicated diagnostic severity level 1, 34.9% (52/149) indicated level 2, and 24.2% (36/149) indicated level 3. Across both diagnostic domains, a slight majority of the participants were identified as level 1.

Among the participants included in our dataset, 96.4% (486/504) had at least two administrations of the Vineland-3 that included the internalizing and externalizing subscales of the maladaptive domain. Within the Q-Global assessment administration platform, the maladaptive domain is optional; the clients with missing data for this domain were excluded.

Among the participants in the overall dataset, 85.5% (431/504) had at least one valid CFQL-2 administration on file (administrations were considered invalid if eight or more questions were skipped). To evaluate change in QoL over time, we compared the first and most recent CFQL-2 scores for the 59.1% (298/504) of participants who had at least two valid administrations on file. For this dataset, the CFQL-2 was administered as an interview conducted by the BCBA with the participant’s primary caregiver.

**Figure 1.**
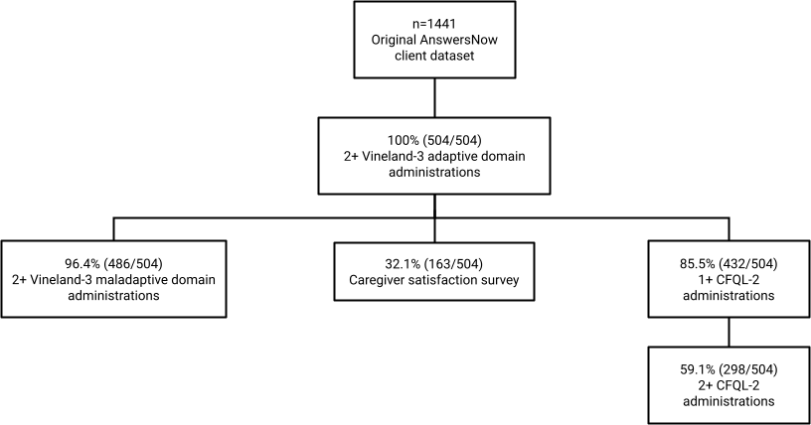
Breakdown of subsamples by measure. CFQL-2: Child and Family Quality of Life, Second Edition.

The final measure evaluated was caregiver satisfaction. Our dataset contained responses from a sample of 32.1% (162/504) of the total dataset. Figure 1 provides a breakdown of the different measures evaluated, the sample sizes for each, and the percentage of the total sample for which that measure was available.

### Service Hours

The average hours of services rendered per week are closely aligned across funding sources, with Medicaid recipients receiving a slightly higher number of hours per week on average 2.66 (SD 1.06) hours compared to non-Medicaid recipients who averaged 2.44 (SD 0.99) hours. That weekly alignment translates to a consistent average of total hours of services rendered, where Medicaid recipients received a slightly higher number of total hours on average (117.14, SD 90.08 h) as compared to non-Medicaid recipients who averaged 116.77 (SD 87.63) hours. The combined averages closely resemble the averages for Medicaid recipients, with an average of 2.6 (SD 1.05) hours per week of services rendered and an average of 117.03 (SD 89.26) total hours rendered. These results are consistent with the total sample size being more heavily comprised of Medicaid recipients.

### Vineland-3 Descriptives

**Figure 2.**
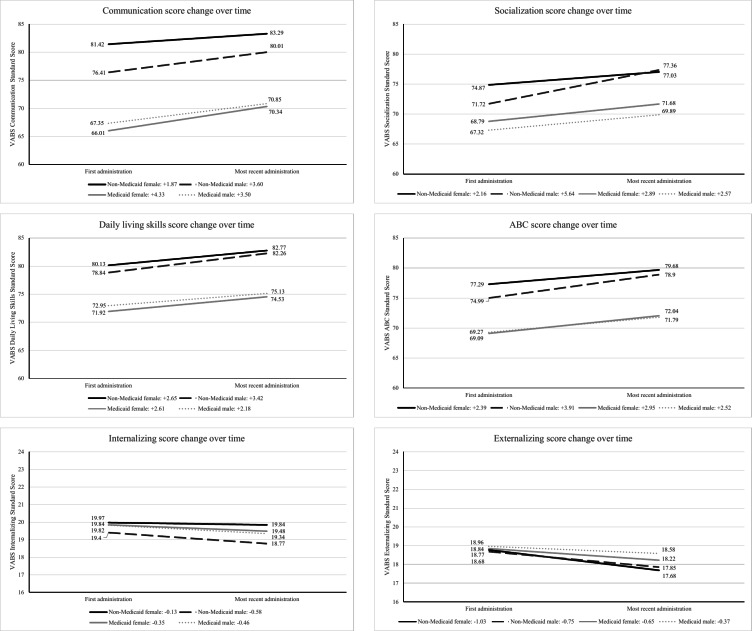
Average domain score and domain score change; first assessment to most recent assessment across Non-Medicaid and Medicaid Recipients. ABC: Adaptive Behavior Composite; VABS: Vineland Adaptive Behavior Scales

The following section is devoted to providing the descriptive statistics associated with the Vineland-3 administrations. Participants ranged from having 2 to 7 Vineland-3 administrations. Figure 2 displays the various score changes between the first assessment administration and the last assessment (most recent) administration for all participants, broken down by funding source and gender, and includes the adaptive behavior domains, the maladaptive behavior domains, and the ABC. The scoring of the Vineland-3 is based on the following recommended interpretation guidelines: high=130‐140, moderately high=115‐129, adequate=86‐114, moderately low=71‐85, and low=20‐70 for scoring the adaptive behavior domains, and average=1‐17, elevated=18‐20, and clinically significant=21‐24 on the maladaptive behavior domains [11].

At the first administration, non-Medicaid funded participants tended to have the highest adaptive behavior domain scores on average, and the averages across both funding groups and genders were consistent in the maladaptive behavior domains. The ideal pattern is for the scores from the adaptive behavior domains to increase across administrations and the scores from the maladaptive behavior domains to decrease. Across both funding groups and genders, there was improvement in all domains over time, suggesting that, on average, participants displayed more adaptive behavior and less maladaptive behavior.

Additionally, further illustrating the change from the first administration to the most recent administration, the average standard score change across domains according to funding source and gender is also included in Figure 2. While there was variability across the domains based on funding source and gender, on average, there were increases in all adaptive behavior domains across gender and funding source, and decreases in the maladaptive behavior domains across gender and funding source, with the least amount of average change being in the maladaptive behavior domains.

Across all adaptive and maladaptive behavior domains, average aggregate standard score changes were positive. Participants’ (n=504) communication standard scores increased an average of 3.56 (SD 9.77) points, socialization standard scores increased an average of 3.34 (SD 11.62) points, daily living skills’ standard scores increased an average of 2.58 (SD 7.89) points, and ABC standard scores increased an average of 2.92 (SD 7.89) points. Within the maladaptive behavior domain, participants’ (n=486) v-scale scores decreased by 0.45 (SD 1.81) points on the internalizing subdomain and by 0.55 (SD 2.05) points on the externalizing subdomain. The results indicate improvements in both the adaptive and maladaptive domains; however, it is worth noting that the SDs for the socialization and daily living skills domains were higher, which indicates greater variability in those domains.

**Figure 3.**
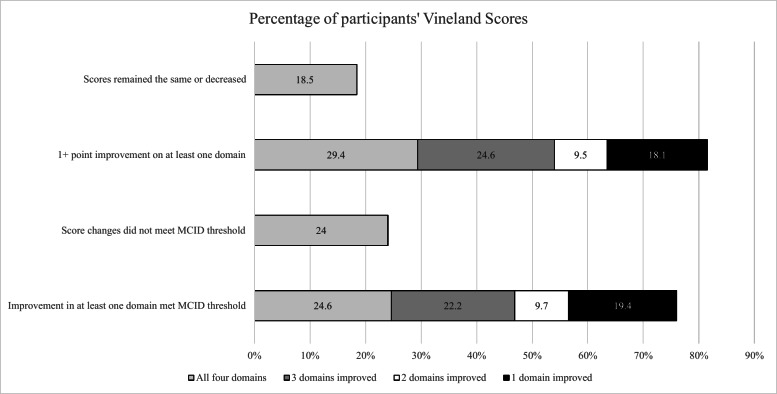
Bar graph of Vineland scores of participants. MCID: minimal clinically important difference.

To further evaluate changes in domain standard scores, the percentage of participants showing a minimum of a 1-point improvement was calculated for the full sample. Figure 3 displays the pattern of standard score changes for the participants in detail, illustrating that 81.5% (411/504) of participants demonstrated a standard score change of +1 point in at least one domain, and 29.4% (148/504) showed a standard score change of +1 point in all domains. Participants who showed either no standard score change or a –1 or greater point change made up 18.5% (93/504) of the sample.

Also depicted in Figure 3 are the percentages of participants who met MCID thresholds for improvement from the first to most recent assessment administration, where the MCID scale is change in communication ≥2.0, change in socialization ≥2.6, change in daily living skills ≥2.6, and change in ABC ≥2.0 [12]. A total of 25% (124/504) of participants met MCID thresholds for all the domains, while 24% (121/504) did not meet MCID thresholds in any of the domains. A total of 76% (383/504) of participants met the MCID threshold for at least one domain. A total of 45% (13/31) of non-Medicaid females met MCID thresholds in all internalizing domains, whereas 57.9% (70/121) of non-Medicaid males met MCID thresholds in ABC, 52.9% (64/121) in communication, 48.8% (59/121) in daily living, and 56.2% (68/121) in socialization. A total of 56% (48/85) of Medicaid recipient females met thresholds in ABC and communication, 48.2% (41/85) in daily living, and 57.7% (49/85) in socialization. The data for Medicaid recipient males indicates that 50.9% (136/267) met thresholds for ABC, 56.9% (152/267) in communication, 43.8% (117/267) in daily living, and 44.9% (120/267) in socialization. Overall, the highest MCIDs were observed in the communication and ABC domains across genders and funding sources.

Additionally, changes in internalizing and externalizing scores are often accounted for in terms of the following clinically significant scoring categories: average=1‐17, elevated=18‐20, and clinically significant=21‐24. Due to the nature of how the score ranges differ across the three categories, substantial movement across categories is less likely between the average category and the other categories because the score range for the average category is much larger than the other two categories.

### Internalizing Scores

In the context of internalizing scores, in the first assessment administration, 11.9% (32/267) of Medicaid recipient males fell into the average category, 59.9% (160/267) into the elevated category, and 37.1% (99/267) into the clinically significant category. At the most recent administration, there was no substantial change in the percentage of Medicaid recipient males falling into any of the three categories. At the first administration, 17.4% (21/121) of non-Medicaid males fell into the average category, while 61.9% (75/121) fell in the elevated category and 34.7% (42/121) in the clinically significant category. Similarly to Medicaid recipient males, at the most recent administration, there was no substantial change in the percent of non-Medicaid recipient males belonging to all three categories.

While the category composition was slightly different for females compared to males, there was generally a similar trend in the category composition from the first administration to the most recent administration in internalizing scores for females as there was for males. At first administration, 16.5% (14/85) of Medicaid recipient females fell into the average category, 42.4% (36/85) in the elevated category, and 47.1% (40/85) in the clinically significant category. At the most recent administration, 16.5% (14/85) were in the average category, 43.5% (37/85) in elevated, and 40% (34/85) in the clinically significant, indicating a 7.1% decrease in the percentage of Medicaid recipient females who fell into the clinically significant category compared to the first administration. At the first administration, approximately 6.5% (2/31) of non-Medicaid recipient females were in the average category, 58.1% (18/31) in the elevated category, and 35.5% (11/31) in the clinically significant category. At the most recent administration, however, 6.5% (2/31) were in the average category, 61.3% (19/31) in the elevated category, and 32.3% (10/31) in the clinically significant category, indicating a slight decrease in the number of females belonging to the clinically significant category.

### Externalizing Scores

The results for externalizing categories generally indicated more pronounced movement as compared to internalizing scores. At the first administration, there were 19.1% (51/267) in the average category, 58.1% (155/267) in the elevated category, and 22.9% (61/267) in the clinically significant category. At the most recent administration, however, 24% (64/267) of Medicaid males were in the average category, 58.8% (157/267) in the elevated category, and 17.2% (46/267) in the clinically significant category. These results indicate a reduction in the number of males in the clinically significant category and an increase in the number of those in the average category. Non-Medicaid males followed a similar but more pronounced pattern with 24.8% (30/121) in the average category, 51.2% (62/121) in the elevated category, and 24% (29/121) in the clinically significant category at first administration. Whereas, at the most recent administration, 35.5% (43/121) were in the average category, 51.2% (62/121) in the elevated category, and 13.2% (16/121) in the clinically significant category. These results indicate an increase in the number of non-Medicaid males in the average category and a 55% reduction in the number of non-Medicaid males in the clinically significant category.

At the first administration, 23.5% (20/85) of Medicaid recipient females were in the average category, 50.6% (43/85) in the elevated category, and 25.9% (22/85) in the clinically significant category. At the most recent administration, 31.8% (27/85) were in the average category, 51.8% (44/85) in the elevated category, and 16.5% (14/85) in the clinically significant category. These results indicate a clear increase in the number of Medicaid females in the average category, a negligible change in the elevated category, and a substantial decrease in the number belonging to the clinically significant category. Non-Medicaid females, however, showed the most significant change from the first administration to the most recent administration. At the first administration, 19.4% (6/31) of the non-Medicaid females were in the average category, 51.6% (16/31) were in the elevated category, and 29% (9/31) were in the clinically significant category. By contrast, at the most recent administration, 38.7% (12/31) were in the average category, 54.8% (17/31) were in the elevated category, and 6.5% (2/31) were in the clinically significant category indicating a 50% (6/12) increase in the number of non-Medicaid females in the average category and a 77.8% (7/9) decrease in the number of non-Medicaid females in the clinically significant category. These results indicate an overall reduction in external maladaptive behavior for males and females across both funding categories.

### Vineland-3 Statistics

Several statistical analyses were conducted to determine the potential relationships between relevant variables and any possible predictive power of outcomes. Those variables included gender, funding source, diagnosis severity levels, treatment modality, and age at first administration. The following section provides a summary of the analyses conducted and the resulting conclusions.

Table 1 illustrates the level of variance associated with each adaptive behavior domain according to funding source type. There is a larger sample size of Medicaid participants vs non-Medicaid participants. Despite that difference, there is a similarity between the two groups outside of the ABC composite score, where there is less variance noted for Medicaid participants. There is, however, a substantial amount of variance for each domain and for each funding source, with the highest levels of variance associated with the socialization domain for both participant groups.

**Table 1.** Variance.

Domain	Medicaid count	Medicaid variance	Non-Medicaid count	Non-Medicaid variance
ABC^a^	352	56.37	152	75.58
Communication	352	93.53	152	100.23
Daily living	352	97.02	152	117
Socialization	352	133.86	152	134.6

a ABC: Adaptive Behavior Composite.

Table 2 displays a summary of the various statistical tests run: *t* test results and Cohen *d* calculations testing for statistical significance across adaptive behavior domains, CIs for domain standard score change, and the *t* test results testing for statistical significance across domains by gender and funding source. There was slight statistical significance for the socialization domain as compared to the other domains (*P*=.04). The socialization domain has the largest effect size of the domains (0.197), but it is still relatively small. The Cohen *d* calculations suggest a smaller and therefore minimal difference across the domains. All domains other than socialization cross zero, further emphasizing the lack of statistical significance. There is a demonstrated statistically significant difference (*P*=.02) between males across funding sources in the socialization domain, however. Males who were supported by non-Medicaid sources showed more significant improvement as compared to males supported by Medicaid. No other gender or funding-related differences were observed.

**Table 2.** Test statistics summary.

Domain	*t* stat^a^ (*df*)	*P* value	Sig^b^ (*P*<.05)	Cohen *d*	95% CI	Male			Female		
						Mean (SD)	Non-M.^c^ mean (SD)	*P* value	Mean (SD)	Non-M. mean (SD)	*P* value
ABC^d^	*−*1.2 (185)	.23	No	0.124	−0.067 to 0.314	2.52 (0.56)	3.91 (1.02)	.13	2.95 (0.77)	2.39 (0.33)	.75
Communication	0.47 (185)	.64	No	−0.046	−0.236 to 0.144	3.49 (0.56)	3.60 (1.02)	.92	4.31 (0.77)	1.87 (0.33)	.32
Daily living skills	−0.96 (185)	.34	No	0.096	−0.094 to 0.287	2.18 (0.56)	3.42 (1.02)	.29	2.61 (0.77)	2.61 (0.33)	.99
Socialization	−2.03 (186)	.04	Yes	0.197	0.007 to 0.388	2.57 (0.56)	5.65 (1.02)	.016	2.89 (0.77)	2.16 (0.33)	.76

a Stat: statistic.

b Sig: significance.

c Non-M.: non-Medicaid.

d ABC: Adaptive Behavior Composite.

Multiple regression analyses were conducted (Table 3). The first regression analysis assessed the relationship between age at first administration and domain standard score change. The results indicate that older age is associated with smaller improvements in domain standard scores. Additionally, statistically significant relationships were found between age at first administration and the ABC (*P*=.03)*,* communication (*P*=.02)*,* daily living skills (*P*=.02)*,* and socialization (*P*=.02) domains. This further emphasizes how one’s age at first administration has some relationship to the extent to which adaptive behavior development occurs in general. It is important to note, however, that ABC is a composite score, so there should be some statistically significant relationship between ABC and the other domains. Finally, the *R*^2^ values are low, which indicates that age at first administration explains only a small portion of the variance in standard score changes.

**Table 3.** Regression analysis summary.

Analysis and predictor, and domain	Coefficient	*P* value	*R*²	95% CI	Significant
Age → score change					
Age at first administration					
Int.^a^	−0.0123	.48	0.008	N/A^b^	No
Ext.^c^	−0.0187	.39	0.011	N/A	No
ABC^d^	−0.0452	.03	0.064	N/A	Yes
Com.^e^	−0.0528	.02	0.072	N/A	Yes
DLS^f^	−0.0496	.02	0.069	N/A	Yes
Soc.^g^	−0.0581	.02	0.078	N/A	Yes
Severity → score change					
SC^h^ severity					
Overall	1.2308	.61	0.008	–3.509 to 5.971	No
RRB^i^ severity					
Overall	−0.967	.69	0.008	–5.708 to 3.774	No
Age and hours → MCID^j^					
Age					
Com.	−0.0087	.68	N/A	N/A	No
Hours					
Com.	0.0009	.51	N/A	N/A	No
Age					
DLS	−0.0031	.55	N/A	N/A	No
Hours					
DLS	0.0008	.55	N/A	N/A	No
Age					
Soc.	−0.0074	.72	N/A	N/A	No
Hours					
Soc.	0.0012	.41	N/A	N/A	No
Age					
ABC	−0.0036	.86	N/A	N/A	No
Hours					
ABC	0.0009	.56	N/A	N/A	No

a Int.: internalized.

b N/A: not available.

c Ext.: externalized.

d ABC: Adaptive Behavior Composite.

e Com.: communication.

f DLS: Daily Living Skills.

g Soc.: socialization.

h SC: social communication.

i RRB: restrictive and repetitive behavior.

j MCID: minimal clinically important difference.

Next, a regression analysis was conducted to assess whether severity level is a predictor of positive domain standard score change (Table 3). The model explains less than 1% of variance in average standard score change (*R*²=0.008). Therefore, the results suggest that severity level is neither statistically significant (*P*=.61) nor is it a strong predictor of overall improvement across domains.

Lastly, a regression analysis (Table 3) was conducted to determine if age and hours of service were predictors of domain MCID thresholds being met. The results suggest that neither age nor hours of service was a significant predictor of MCID improvement (*P*<.05).

With regard to the maladaptive behavior domains (internalizing and externalizing), a paired sample *t* test was conducted for internalizing standard scores and externalizing v-scale scores between first administrations and most recent administrations. The internalizing standard scores from the first Vineland-3 administration were a mean of 9.73 (SD 1.96), and the most recent administration results were a mean of 19.28 (SD 2.07). This yielded an analysis result of *t*_485_=5.43, *P*<.001. The change in internalizing standard scores from the first administration to the most recent administration is statistically significant (*P*<.001). The decrease in mean indicates a decrease in internalizing standard scores overall, which suggests improvement (reduction) in maladaptive internalizing behaviors between administrations.

With regard to externalizing standard scores, the paired sample *t* test comparing externalizing scores from the first Vineland-3 administration to scores from the most recent Vineland-3 administration was mean 19.37 (SD 1.79) for the first administration, and mean 18.96 (SD 1.93) for the most recent administration. This yielded an analysis result of *t*_485_=6.01, *P*<.001. The change in externalizing v-scale scores from the first administration to the most recent administration is highly statistically significant (*P*<.001). The decrease in mean indicates a decrease in externalizing standard scores overall, which suggests improvement (reduction) in maladaptive externalizing behaviors between administrations.

### CFQL-2 Descriptives

This section is devoted to providing descriptive statistics for CFQL-2 administrations. Of the total sample of 504 participants, 431 (85.5%) participants received at least one CFQL-2 administration. Some participants received treatment before AnswersNow, Inc, adding the CFQL-2 to its assessment battery, while other participants did not have the CFQL-2 administered for clinical or operational reasons. Most participants (231/431, 53.6%) were males who received support from Medicaid. Medicaid-funded participants also accounted for the majority (304/431, 70.1%) of the total sample (n=431). The process of evaluating CFQL-2 results primarily focused on assessing total QoL and changes in total score.

The scores for each CFQL-2 scale across all participants (n=431) at the most recent administration were child QoL (mean 12.9, SD 2.57; range 0‐20)*,* family QoL (mean 13.3, SD 4.2; range 0‐20)*,* caregiver QoL (mean 14.1, SD 3.82; range 0‐20)*,* financial QoL (mean 11.3, SD 2.86; range 0‐15), social network QoL (mean 15.1, SD 3.44; range 0‐20)*,* partner network QoL (mean 11.5, SD 7.64; range 0‐20)*,* and coping QoL (mean 11.7, SD 2.31; range 4‐15). Total QoL score mean at most recent administration was 92.4 (SD 16.13; range 51‐125).

Table 4 represents the percentage of participants’ QoL scaled scores where improvement occurred, as well as the type of change that occurred based on the most recent CFQL-2 administration. Improvement was determined based on the following scale thresholds: child QoL, family QoL, caregiver QoL, social network, and partner network QoL (high=16‐20, adequate=8‐15, and low=0‐7); financial QoL (high=12‐15, adequate=6‐11, and low=0‐5), and coping QoL (high=12‐15, adequate=8‐11, and low=4‐7). Overall, 40.9% (122/298) of participants showed an increase in total QoL, 32.9% (98/298) remained the same, and 25.8% (77/298) decreased, with 72.1% (215/298) of the participants experiencing adequate QoL according to the assessment.

**Table 4.** Percentage of participants showing improvement and directional change at the most recent administration.

	Improvement scale thresholds			Change direction		
	High	Adequate	Low	Same	Increased	Decreased
Child	15.31	81.67	2.32	43.62	34.11	22.27
Family	35.03	51.04	12.99	42.23	37.12	20.65
Caregiver	38.05	56.38	3.94	42.46	35.96	21.58
Financial	69.61	22.04	7.42	51.74	28.77	19.49
Social network	0	0	0	46.4	25.99	27.61
Partner relationships	0	0	0	58.47	17.63	23.43
Coping	72.85	25.06	2.09	45.48	31.09	23.43
Total	27.84	71.69	0.46	33.18	40.6	26.22

### CFQL-2 Statistics

Several statistical analyses were conducted to determine the potential relationships between relevant variables and any possible predictive power of the CFQL-2 outcomes. Those variables included gender, funding source, diagnosis severity levels, and age at the first administration. The following section provides a summary of the analyses conducted and the resulting conclusions.

Table 5 displays the results of a regression analysis testing whether age at first administration, Medicaid status, ASD severity level, initial total QoL, average service hours per week, or gender are significant predictors of change in total QoL standard scores. The results indicate that age at first administration (*P*=.95)*,* Medicaid status (*P*=.58), ASD severity level (*P*=.55)*,* average number of service hours per week (*P*=.41), and gender (*P*=.56) were not significant predictors of change in the average total QoL score. Initial total QoL (ie, the first total QoL score captured), however, was a significant predictor (*P*<.001) of change, indicating that the higher the initial total QoL score, the less likely the participant will be to show improvement in scores over time and the more likely a participant with a lower initial total QoL score would be to show improvement.

**Table 5.** Regression analysis of predictors of clinically significant changes in total QoL^a^ scores.

Predictor	OR^b^ (CI)	*P* value
Intercept	25.15 (1.12 to 565.21)	.04
Age at first administration	1 (0.92 to 1.1)	.95
Medicaid status	1.25 (0.56 to 2.83)	.58
ASD^c^ severity level	1.17 (0.71 to 1.92)	.55
Initial total QoL score	0.96 (0.93 to 0.98)	.001
Average hours per week between	1.17 (0.81 to 1.71)	.41
First and most recent administration	1.17 (0.81 to 1.71)	.41
Gender	1.3 (0.54 to 3.11)	.56

a QoL: quality of life.

b OR: odds ratio.

c ASD: autism spectrum disorder.

Additionally, a chi-square test of independence was conducted to examine the relationship between Medicaid status and total QoL score. The results indicated that 127 of 211 (60.2%) Medicaid participants experienced positive changes, and 48 of 87 (55.2%) of non-Medicaid participants experienced positive changes. The difference between the two groups, however, was not statistically significant, *χ²*_1_=0.5, *P*=.50, indicating that Medicaid status was not associated with a higher likelihood of positive total QoL change.

### Caregiver Satisfaction

An optional 4-question Likert scale–based caregiver satisfaction survey was conducted to capture more anecdotal information related to caregiver experience and provide a mechanism for caregivers to give feedback regarding services. Of the 504 participants in the sample, 32% (n=162) completed the satisfaction survey. The survey included the following questions and response options related to satisfaction and experience: (1) how likely are you to recommend services to a family with a child similar to yours (scored on a scale of 1‐10 where 1=not at all likely and 10=extremely likely), (2) have services been effective for your child (with the answer options of very effective, effective, unsure, ineffective, or very ineffective), (3) what aspect of services is most important to you (with the answer options of accepts my insurance, caregiver training, effective therapies, experienced BCBAs, no waitlist, virtual or video format, all the above, or other), and (4) in the last month, how many times have you considered moving your child to residential services and/or calling emergency services to support your child (with the answer options of never, 1‐2 times, or 3+ times).

The responses to the questions provide a better understanding of how the caregivers who responded view the services they are receiving. A large portion of the respondents indicated being extremely likely (136/162, 84%) to recommend services, with only 16% (26/162) answering the question with a score of 9 or less. An overwhelmingly large portion of caregivers indicated services were very effective or effective (154/162, 95.1%), with only 2% (3/162) indicating services were ineffective or very ineffective. Just under half the respondents (66/162, 40.7%) indicated effective therapies as the most important aspect of services, followed by the virtual or video format (34/162, 21%) and having experienced BCBAs (32/162, 20%), with only 17.9% (29/162) of respondents indicating caregiver training, accepts my insurance, no waitlist, all the above, and other combined. Lastly, 93.8% (152/162) of respondents indicated they had never considered moving their child to residential services and/or calling emergency services, with 3.7% (6/162) indicating they had considered the options 1‐2 times, and 1.8% (3/162) indicating they had made those considerations 3 or more times.

A chi-square test of significance (*χ²*_3_=31.9, *P*=.60; n=162) was conducted to assess the relationship between positive change in Vineland-3 scores across domains and caregiver satisfaction. There was no significance between Vineland-3 domain score change and caregiver satisfaction (*P*=.52). In addition, a chi-square test of significance (*χ²*_21_=2116.6, *P*=.74; n=162) was conducted to evaluate the association between caregiver satisfaction and which aspect of service was indicated as being most important. There was no statistically significant association between caregiver satisfaction and which aspect of services was indicated as being most important (*P*=.76). Last, a chi-square test of significance (*χ²*_6_=623.8, *P*<.001; n=162) was conducted to assess the relationship between caregiver satisfaction and frequency of considering residential or emergency services. The results indicate there is a significant statistical association between caregiver satisfaction and the frequency of considering residential or emergency services (*P*<.001). These results suggest that families who considered crisis interventions more frequently are more likely to report lower satisfaction with the services being received, and those who did not consider crisis interventions are more likely to report higher satisfaction with the services being received.

## Discussion

### Principal Findings

This study was designed to evaluate the acceptability and feasibility of a novel approach to delivering ABA services, specifically a fully virtual, BCBA-delivered, focused ABA service model for individuals diagnosed with ASD. This study explored caregiver perceptions of service effectiveness and satisfaction, which indicate high family satisfaction and positive caregiver perceptions of effectiveness. We also found that participants’ adaptive functioning, maladaptive behaviors, and QoL improved between the first assessment battery and the most recent assessment battery according to standard assessments. Given the retrospective design of this study, these changes are being interpreted as changes over time during service delivery and not the direct results of treatment.

Prior research on comprehensive, in-person ABA interventions generally shows that participants demonstrate adaptive behavior improvements, as measured by the Vineland-3, through participation in those programs. For example, Aitken et al [15] found that out of a sample of 1225 children diagnosed with ASD who participated in comprehensive in-person services, 61% demonstrated improvement across multiple adaptive domains of the Vineland-3. Similarly, Adelson et al [14] observed improvements in all Vineland-3 adaptive domains and both maladaptive subdomains. In the present study, the greatest improvements were observed in communication and socialization; however, 81.5% (411/504) of participants exhibited at least a one-point improvement in one or more adaptive behavior domains, and 29.4% (148/504) improved across all adaptive behavior domains. In the maladaptive behavior domains, 38.1% (185/486) of participants showed improvement in the internalizing domain, and 34% (165/486) showed improvement in the externalizing domain. These outcomes translate to significant reductions in either the frequency, intensity, and/or duration of maladaptive behavior observed over time. Furthermore, 76% (383/504) of participants met MCID thresholds in at least one domain. The results indicate the feasibility of this virtual-focused service model as an option for families seeking services to address adaptive behavior deficits and challenging behavior excesses.

QoL changes, as measured by the CFQL-2, were also encouraging. Most participants (175/298, 58.79%) showed improvement in overall QoL scores over time. Previous research has demonstrated the positive impact of caregiver involvement in services on parental self-efficacy and parental stress [30] as well as QoL within the family system [14]. Families in the present study participated in caregiver training, which could be correlated with improvements in QoL observed from the first to the most recent CFQL-2 administration. Regression analyses indicated that initial (baseline) QoL scores were predictive of improvement, with lower baseline scores associated with greater gains. This finding suggests that individuals with less adaptive behavior and lower perceived QoL at the outset of therapy may experience more pronounced benefits from intervention over time as compared to those with a more robust adaptive behavior repertoire and higher perceived QoL at the outset. Recent systematic reviews and meta-analyses examining the role of a child’s age, baseline cognitive ability, and the amount of intervention received suggest findings across studies remain inconsistent and advocate the need for further research with controlled designs to better understand how preprogram characteristics are associated with intervention-related outcomes [34,35]. Future studies should further analyze these variables using a comparison group to better evaluate the differential impact of services across groups.

Caregiver satisfaction data revealed overwhelmingly positive opinions of service effectiveness, with 95.1% (154/162) of respondents rating services as effective or very effective and 84% (136/162) reporting they were extremely likely to recommend services. However, exploratory analyses examining associations between satisfaction and other variables yielded mixed results. No significant relationship was found between caregiver satisfaction and Vineland-3 score improvements or between caregiver satisfaction and the aspect of services the caregivers identified as being most important. These findings suggest that caregiver satisfaction may be additionally influenced by factors beyond measurable clinical outcomes, such as communication quality, accessibility, or perceived responsiveness of providers.

Conversely, a significant association between caregiver satisfaction and the frequency of caregivers considering residential or emergency services (*χ*²_6_=23.82, *P*<.001; Cramér *V*=.27; n=162) did emerge. Families who reported considering crisis interventions more frequently were also more likely to express lower satisfaction with services, whereas those who never considered such interventions reported higher satisfaction with services. This moderate effect size underscores the importance of addressing caregiver stress and crisis risk as part of service delivery, as these factors may substantially impact overall satisfaction and engagement throughout the course of therapy.

Previous studies have found similar results when evaluating the feasibility and acceptability of behavior-analytic virtual services for autistic children. Simcoe et al [36] evaluated a treatment model in which a trained consultant (ie, BCBA, speech and language pathologist, or early childhood educator) supervised early intervention providers who delivered caregiver coaching in naturalistic developmental behavioral interventions either in-person or virtually. The researchers evaluated three different caregiver- and consultant-completed child outcome measures, as well as acceptability of treatment (caregivers and early intervention providers). They found improvement across all child outcome measures and high acceptability among caregivers and consultants. Similarly, a 2019 review study examined the results of 28 different studies evaluating telehealth as a treatment model for delivering behavior analytic services to individuals on the autism spectrum and found that telehealth can be an acceptable model for these services [32].

This study extends the existing literature by specifically evaluating a fully virtual, BCBA-delivered, focused ABA therapy model for autistic individuals. This study demonstrated the feasibility of implementing this novel treatment model at scale by analyzing data for 504 distinct clients who had at least two Vineland-3 administrations on file. While most ABA therapy programs take a comprehensive approach and require high weekly hours, this study shows that lower dosage focused programs delivering an average of 2.6 hours per week can be acceptable to families seeking support, and that families report positive changes in behavior and QoL over time during service delivery. Finally, this study is aligned with previous research demonstrating that caregiver involvement in therapy is associated with positive behavior change. Taken together with prior research, this study supports the growing body of evidence suggesting the feasibility and acceptability of virtual ABA therapy services. Given the potential challenges associated with in-person, RBT-implemented, comprehensive services some families face, such as lack of access, limited appropriateness of the model for some autistic individuals, lesser qualified providers delivering the majority of services, and high costs, it is important to continue studying and evaluating the impacts of this novel therapy model.

### Limitations

This study was descriptive in nature and relied on deidentified archival data, thus limiting our ability to control for potentially confounding variables such as the specific behavior interventions used, socioeconomic status, comorbid conditions, or caregiver stress levels. Given the descriptive nature of this study, we are unable to determine whether the treatment itself produced improvements in adaptive functioning, maladaptive behaviors, or QoL. Demonstrated improvements could be attributed to maturation effects, regression to the mean, or other causes outside the scope of the services delivered. Similarly, the absence of a comparison group limits our ability to draw direct conclusions about the effectiveness of the fully virtual, focused service model compared to that of traditional in-person comprehensive service models.

Some aspects of the current sample also limit the generalizability of the observed changes in adaptive behaviors, maladaptive behaviors, and QoL, in addition to the levels of caregiver satisfaction. One such aspect is the limited geographic representation within our sample. Participants reside across five states, with most participants concentrated in just two states. In addition, diagnostic severity level was only available for a small subset of the total sample, making the composition of the full sample unclear. Taken together, these two features of the current sample suggest cautious interpretation of the generalizability of the findings.

Another important limitation of the analytic sample was that it was derived from a broader clinical population that received services concurrently. The included participants were those who had research consent on file, at least two valid Vineland-3 assessments, a minimum interval between assessments, and ongoing service exposure. Similarly, CFQL-2 and caregiver satisfaction data were available for even smaller subsets of the clinical population. Individuals excluded based on these criteria may have received a qualitatively different experience; it is possible that families who met criteria for inclusion tended to be more engaged, participated longer in services, or had more favorable outcomes than those who were excluded. This further limits the interpretation and generalizability of the data.

An additional limitation of this study is the heterogeneity of the services participants received during treatment. Participants in this study experienced variability in the number of hours received, the ratio of hours by service type, the specific behavior-analytic procedures implemented, and the time between the first and most recent Vineland-3 administrations. We also did not have a direct measure of caregiver engagement or individual goals or goal mastery, both of which were individualized to each participant. Further, there are potential biases associated with the outcome measures evaluated. Specifically, Vineland-3 scores were based on caregiver report; in some cases, a different caregiver completed subsequent Vineland-3 administrations than the caregiver who completed the baseline administration. The CFQL-2 was administered as an interview by the treating BCBA, and satisfaction data were collected voluntarily. These factors introduce a level of variability and potential bias that limit our ability to make direct conclusions about the impact of the services delivered on changes in behavior and QoL. It is possible that some observed differences among participants could be attributed to specific aspects of their treatment that were different from the treatment other participants received. It is also possible that these measurement biases could have resulted in apparent changes in behavior and QoL that were related to momentary caregiver perception rather than true change.

Next, the lack of clear industry benchmarks or MCIDs for the Vineland-3 and CFQL-2 makes evaluation of changes over time difficult. The MCIDs established for the Vineland-II have been used by some existing research, but it is unclear to what extent these MCIDs apply to the Vineland-3. Similarly, there are no established thresholds for clinical significance when evaluating QoL over time using the CFQL-2, making it difficult to determine the extent to which improvements are meaningful.

Finally, there are limitations with the extent to which we evaluated caregiver satisfaction and the specific scores used for evaluating change on the Vineland-3. Specifically, the caregiver satisfaction survey was completed by only a subset of participants who represented less than half the sample of participants (162/504, 32.1%). Representation from the total dataset was limited by 2 factors: (1) the first caregiver satisfaction survey was sent in November 2023, at which time some families had already exited treatment, and (2) participation in the caregiver satisfaction survey was voluntary. This reality introduces potential response bias depending upon the nature of those who completed the survey and their histories with other services, existing familiarity with ABA methodology, and their familiarity with alternative service delivery options. With regard to the Vineland-3 evaluation, we analyzed standard score changes; however, there are scores that may be more sensitive to change over time and therefore better for longitudinal tracking. For example, the growth scale values monitor a person’s progress in the same domain across multiple assessments, vs comparing standard scores from an individual’s performance to that of same-age peers within a domain.

### Implications for Practice

While this study’s design substantially limits causal inference, the findings do highlight the need for further exploration of this novel treatment model in clinical practice. Fully virtual, focused, BCBA-delivered interventions may offer a viable alternative to traditional high-intensity, center-based comprehensive models, particularly for families seeking flexible and/or targeted support or those who have difficulty accessing therapy. The strong caregiver satisfaction ratings suggest that fully virtual focused services can address family needs. Given the strong caregiver satisfaction observed, it is possible that this model could have a multifaceted impact on the socially significant behaviors that affect the independence, well-being, and individual success of those diagnosed with ASD and their families.

The significant association between satisfaction and crisis considerations highlights the need for integrated caregiver support strategies, including proactive crisis planning, coordination of care with a family’s other providers, and stress management resources. Families who are facing crises with their children potentially require support beyond what is typically made available by ABA service providers. It is plausible that a more BCBA-directed and implemented model of services could provide that level of necessary proactive crisis planning and stress management by having the luxury of more frequent connections being created in a more targeted manner. By working directly with caregivers to decrease maladaptive behavior as the primary objective of intervention programming, the BCBA’s support may more meaningfully improve the family’s experience with services as well as the family’s general QoL.

### Future Directions

A great deal of research addressing the role, experience, and needs of the caregiver in the context of participation in ABA service models is needed. For example, research could look to provide deeper insight into caregiver perceptions and to more specifically identify factors that drive satisfaction beyond a child’s progress alone. Future caregiver research might more closely examine the relation between caregiver satisfaction and treatment fidelity, caregiver satisfaction and service duration (eg, do satisfied caregivers stay in treatment longer?), and the level of caregiver involvement and client outcomes. Exploring interventions that specifically target caregiver stress and crisis prevention within remote service frameworks may enhance both satisfaction and overall treatment frequency and efficacy.

Greater emphasis could be placed on examining the combinations of adaptive and maladaptive skills at therapy onset that equate to specific outcomes, rather than more commonly examined variables such as age, gender, diagnostic severity level, and treatment intensity as the primary predictors of outcomes. Additionally, it is feasible to consider the implications of how types of treatment goals, the density of treatment goals, and mastery of goals impact the development of adaptive behavior and the reduction of maladaptive behavior over the course of intervention programming. The relationship among these factors alone could have a direct influence over the Vineland-3 and the CFQL-2 scores, as well as the types of intervention outcomes achieved. Lastly, as noted as a study limitation, growth scale values may be more sensitive to change over time and therefore more effective at longitudinal tracking as opposed to relying on standard scores and/or standard score changes as the primary tracking mechanisms.

Given the difference in treatment plan compositions between comprehensive and focused ABA therapy services, the Vineland-3 ABC standard score may be more appropriate for evaluating improvements in adaptive behavior that result from participation in a comprehensive treatment model. In a focused treatment model, we may expect more targeted gains, with scores in other domains remaining the same or decreasing over time. It would be valuable to assess the extent to which focused services produce improvement in the specific domains directly related to the participant’s treatment focus, and whether those gains are maintained over time when treatment shifts to a different focus. This study described a novel model for delivering ABA services, specifically fully virtual, BCBA-delivered, focused treatment, and evaluated changes in behavior and QoL over time, as well as caregiver satisfaction using a real-world clinical dataset. Future research should compare fully virtual ABA services with those of traditional delivery models through an examination of clinical outcomes, caregiver preferences, and BCBA experiences. Longitudinal studies are needed to assess the durability of adaptive skill gains and QoL improvements over time for recipients of both types of service models, and the inclusion of a comparison group would improve experimental control, allowing for a more robust interpretation of results. Fully virtual, BCBA-delivered, focused ABA services appear to be acceptable and feasible for the families in this study. For some families, these services may address a broad range of needs in an ostensibly more economical and accessible fashion than comprehensive in-person services, without potentially sacrificing the quality of services provided or lessening the socially significant impact on the lives of children and families that the practice of ABA has become synonymous with providing.

## Abbreviations

ABA: applied behavior analysis
ABC: Adaptive Behavior Composite
AFLS: Assessment of Functional Living Skills
ASD: autism spectrum disorder
BCBA: Board Certified Behavior Analyst
CFQL-2: Child and Family Quality of Life, Second Edition
FAST: functional assessment screening tool
HIPAA: Health Insurance Portability and Accountability Act
ICHOM: International Consortium for Health Outcomes Measurement
MCID: minimal clinically important difference
QABF: questions about behavior function
QoL: quality of life
RBT: Registered Behavior Technician
VB-MAPP: Verbal Behavior Milestones Assessment and Placement Program
Vineland-3: Vineland Adaptive Behavior Scales-Third Edition
